# Interferon-free therapy with direct acting antivirals for HCV/HIV-1 co-infected Japanese patients with inherited bleeding disorders

**DOI:** 10.1371/journal.pone.0186255

**Published:** 2017-10-18

**Authors:** Haruka Uemura, Kunihisa Tsukada, Daisuke Mizushima, Takahiro Aoki, Koji Watanabe, Ei Kinai, Katsuji Teruya, Hiroyuki Gatanaga, Yoshimi Kikuchi, Masaya Sugiyama, Masashi Mizokami, Shinichi Oka

**Affiliations:** 1 AIDS Clinical Center, National Center for Global Health and Medicine, Tokyo, Japan; 2 Center for AIDS Research, Kumamoto University, Kumamoto, Japan; 3 Genome Medical Sciences Project, Research Institute, National Center for Global Health and Medicine, Chiba, Japan; Kaohsiung Medical University, TAIWAN

## Abstract

**Introduction:**

Almost 30 years ago, about 30% of Japanese hemophiliacs became infected with HIV-1 and hepatitis C virus (HCV) after receiving contaminated blood products. While several studies have reported the high efficacy and safety of direct acting antivirals (DAA) in HIV-1 co-infected patients, such data are limited in hemophiliacs.

**Methods:**

We conducted a single-center, open-label study involving 27 Japanese patients (median age; 45 years) with inherited bleeding disorders who were co-infected with HCV/HIV-1. Patients with HCV genotype 1 (GT1) and GT4 received ledipasvir (90 mg) plus sofosbuvir (400 mg), those with HCV GT2 received sofosbuvir plus weight-based ribavirin, and those with HCV GT3 received daclatasvir (60 mg) plus sofosbuvir. Treatment was continued for 12 weeks in all patients. The primary endpoints were rate of sustained virologic response at 12 weeks after end of therapy (SVR12) and occurrence of adverse events during DAA therapy.

**Results:**

Eighteen (67%) patients had had received interferon-based therapy, and 11 (41%) had compensated cirrhosis. HCV genotypes were GT1a 4 (15%), GT1b 16 (59%), GT1 undetermined 2 (7%), GT2a 1 (4%), GT3a 3 (11%) and GT4a 1 (4%). All patients were on combination antiretroviral therapy (cART) and had undetectable HIV-1 viral load (<20 copies/μL) at baseline. All patients achieved SVR12. Serious adverse events were observed in 3 patients: arteritis of the leg, which resolved after completion of DAA therapy, asymptomatic QT prolongation and gastrointestinal hemorrhage. cART failure was noted in one patient due to emergence of raltegravir resistance during ledipasvir/sofosbuvir treatment. Although α-fetoprotein, Mac-2 binding protein glycosylation isomer (M2BPGi), and Fibro Scan (FS) scores decreased in most patients during DAA therapy, M2BPGi (>2.0 cutoff index) and FS scores (>15.0 kPa) were still high in 6 patients at week 36.

**Conclusions:**

DAA therapy is effective in all patients. However, adverse events and efficacy of cART should be monitored closely.

## Introduction

Almost all and around 30% of Japanese hemophiliacs were infected with hepatitis C virus (HCV) and HIV-1, respectively, via contaminated blood products, more than 30 years ago [1, 2]. Because our institution (AIDS Clinical Center at National Center for Global Health and Medicine) was established in 1997, mainly for the management of patients infected with HIV-1 via contaminated blood products, many of the above patients visit our facility on a regular basis up to the time of writing of this paper.

It is believed that HCV disease progression in HIV-1 co-infected patients is faster than that of HCV mono-infected patients [3]. In fact, liver cirrhosis has already developed in some of the above Japanese patients [4]. In the era of combination-active antiretroviral therapy (cART), the main cause of death in Japanese co-infected hemophiliacs is not related to HIV-1 but rather to HCV-related diseases [2]. Several studies have reported the high efficacy and safety of direct acting antiviral (DAA) therapy in HIV-1 coinfected patients [5–9]. Although two studies reported the high efficacy and safety of DAA therapy in HIV-1 coinfected hemophiliac patients (by contaminated blood products), the effect of such therapies in hemophiliacs is limited [10, 11]. Most of the Japanese hemophiliacs have received various forms of therapies against HIV-1, starting with AZT monotherapy, dual nucleoside reverse-transcriptase inhibitors (NRTIs) therapy, and cART, and some have histories of treatment failures. Some of these patients have already developed drug resistance mutation against NRTIs while in some the cART resulted in suppression of plasma viral load to undetectable levels [12]. Accordingly, monitoring of the efficacy of current cART during DAA therapy for HCV treatment should be clinically interesting.

Previous studies reported progression of liver fibrosis and development of hepatic cellular carcinoma (HCC) after interferon-induced eradication of HCV [13, 14]. On the other hand, the effect of DAA on cancer suppression is unknown at present. Thus, it is important to assess the progression to liver fibrosis and HCC after eradication of HCV with DAA [15], especially in hemophiliac patients with long history of HCV infection.

The present study was designed to determine the efficacy and safety of DAA therapy combined with cART in HCV/HIV-1 co-infected patients with inherited bleeding disorders. We also investigated changes in markers of fibrosis and tumorigenesis during and after DAA therapy.

## Methods

### Patients

We conducted a single-center, open-label study involving Japanese patients with inherited bleeding disorders with history of co-infection with HCV/HIV-1. All participating patients were co-infected with HCV/HIV-1 through contaminated blood products before 1986 when use of untreated blood products was inhibited. Patients infected with any HCV genotype were included in this study. Patients with renal impairment (eGFR <30 mL/min/1.73 m^2^) and with decompensated cirrhosis, represented by Child-Pugh score higher than 7, were excluded. HCV RNA was still detected in plasma samples of 34 patients who continued to visit our clinic in September 2015. Of these, we enrolled 27 patients (25 males and 2 females) who fulfilled the inclusion and exclusion criteria set out for this study conducted between September 2015 and June 2016.

Japanese hemophiliacs with HIV-1 infection have been treated with antiretroviral drugs since early 1990s. In our cases, all patients except one (96%) had experienced cART switch due to HIV treatment failure or emergence of side effects during the past treatment. The median number of cART switch was six times. In these 27 patients, 16 different cART regimens were used at the time of study, including FTC/TDF (emtricitabine/tenofovir) plus DTG (dolutegravir) in 6 patients, 3TC/ABC (lamivudine/abacavir) plus DTG in 4, 3TC/ABC plus ETR (etravirine) plus RAL (raltegravir) in 2, FTC/TDF plus RAL in 2, and RPV (rilpivirine) plus DTG in 2 (**S1 Table**).

The investigators and all other project personnel are responsible for the conduct of this research protocol in accordance with the ethics principals outlined in the Declaration of Helsinki and Ethical Guidelines for Medical and Health Research Involving Human Subjects published by the Ministry of Health, Labour, and Welfare, Japan. All patients provided written informed consent on participation in the study. The ethics committee approved the off-label use of DAA in this study on October 20, 2015 (NCGM-G-001892, **S1 and S2 Protocols**). The study was also registered in UMIN-CTR on November 6, 2015 (UMIN000019659). In patients infected with GT1, GT2, and GT4, to hasten the initiation of treatment taking into consideration their liver function status, we started the recruitment of patients after the submission of the study protocol, and the approval of this study by the ethics committee was delayed till after the initiation of the recruitment. In patients infected with HCV GT3, because DAA was off-label used, we started the recruitment of these patients after the study registration at the UMIN. The authors confirm that all ongoing and related trials for this drug/intervention are registered.

### Treatment regimen

Patients with HCV genotype 1 (GT1) and GT4 received ledipasvir (90 mg) plus sofosbuvir (400 mg) in a fixed-dose tablet. Patients with HCV GT2 received sofosbuvir (400 mg) plus weight-based ribavirin. Patients with HCV GT3 received daclatasvir (60 mg) plus sofosbuvir (400 mg). The treatment regimen was similar irrespective of the presence or absence of compensated cirrhosis. Treatment was continued for 12 weeks in all patients.

### Study assessment

Physical examination and laboratory tests, including HCV RNA, were performed at screening, baseline, and weeks 2, 4, 8, 12, 24, and 36 after the start of treatment. HCV RNA was measured with the COBAS® TaqMan® HCV test, version 2.0 (Roche Molecular Systems). Virologic response was defined as undetectable HCV RNA (<15.8 IU/mL). All adverse events were graded according to the Division of AIDS Table for Grading the Severity of Adult and Pediatric Adverse Events version 1.0 [16]. Baseline laboratory tests included HCV genotyping and *IL28B* rs12979860 genotyping [17, 18]. Clinical laboratory tests included urine, electrocardiogram, serum levels of Mac-2 binding protein glycosylation isomer (M2BPGi), a marker of liver fibrosis, and α-fetoprotein (AFP) a marker of HCC. M2BPGi score was measured with the use of a fully automated apparatus (HISCL-5000; Sysmex, Hyogo, Japan). Fibro Scan (FS) was measured at baseline, and weeks 4, 8, 12, 24, and 36 after the start of treatment. Ultrasonography and enhanced CT scan were performed at baseline and at week 24 or 36. Patients were followed-up after the day of enrollment till March 2017.

### Endpoints

The primary endpoints of DAA therapy were the rate of sustained virologic response at 12 weeks (after the end of therapy, SVR 12) and occurrence of adverse events. The secondary endpoints were assessment of the delta changes in markers of liver fibrosis and HCC between baseline and 36 weeks. Safety and efficacy of cART during DAA therapy were also investigated.

### Statistical analysis

Comparisons of the mean values were conducted by the paired t test with 2-tail *P* value. Differences were considered statistically significant at *P*<0.05. Statistical analysis was performed using SPSS version 23.0 for Windows (IBM). Confidence intervals were computed using GraphPad Prism 6 for Windows (GraphPad Software).

## Results

### Patients' characteristics

**Fig 1:**
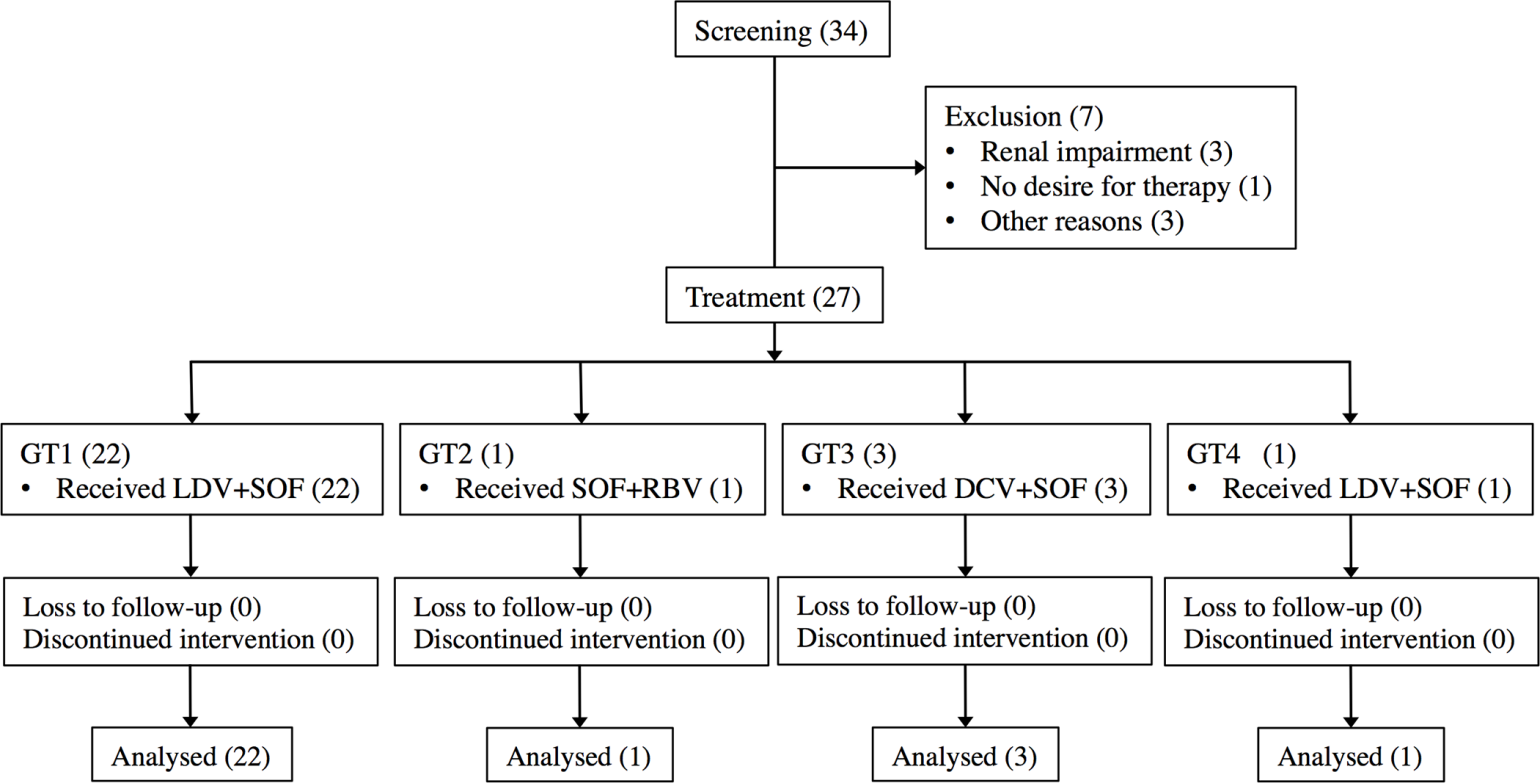
Selection of patients and treatment regimen. Among the 34 patients, 7 were excluded due to the indicated reasons. “Other reasons” are hidden to protect patients’ privacy. The treatment regimen was selected based on the genotype. GT: genotype, (n): number of patients, LDV: ledipasvir, SOF: sofosbuvir, DCV: daclatasvir, RBV: weight-dosed ribavirin.

**Fig 1**illustrates the patient selection process and **Table 1**lists the clinical characteristics of the patients. The median age of the patients was 45 years. The majority had hemophilia A (85%); 18 patients (67%) had undergone interferon-based therapy, 11 patients (41%) had compensated cirrhosis, determined by FS score (>12.5 kPa), together with an aspartate aminotransferase (AST) to platelet ratio index (APRI) of >2 (treatment naïve, 18%; treatment experienced, 82%). HCV genotypes were GT1 in 22 patients (59%), GT2 in 1 (4%), GT3 in 3 (11%) and GT4 in 1 (4%). All patients were on cART and had an HIV RNA level of <20 copies/mL at baseline. The median CD4^+^ lymphocyte count was 467/μL.

**Table 1:** Demographic and clinical characteristics of patients at baseline*.

N	27
Age, median (IQR), years	45 (43–48)
Male, n (%)	26 (96%)
Race, n (%)†	
Asian	27 (100%)
Inherited bleeding diseases, n (%)	
Hemophilia A	23 (85%)
Hemophilia B	2 (7%)
von-Willebrand disease	2 (7%)
Body mass index, median kg/m^2^ (IQR)‡	21.4 (19.0–22.8)
HCV genotype	
1	22
1a	4
1b	16
1 undetermined	2
2	1
3	3
4	1
HCV-RNA, median (IQR), log_10_ IU/mL	6.1 (5.7–6.5)
IL28B rs12979860 genotype, n (%)	
CC	18 (67%)
CT	7 (26%)
TT	2 (7%)
Compensated cirrhosis	11(41%)
Anti-HCV treatment, n (%)	
Naïve	9 (33%)
Past IFN-based therapy	18 (67%)
CD4+ count (IQR), median, cells/μL	467 (325–591)

*IQR interquartile range, HCV hepatitis C virus, and IFN interferon.

†Race was self-reported.

‡Body-mass index = weight (in kg) / (height in m)^2^.

### Virologic response to DAA treatment

All patients completed the 12-week treatment. The HCV RNA level became undetectable by week 8 in all patients. All patients achieved SVR12 (confidence interval 87.2% to 100%). We also confirmed that all patients achieved SVR24.

### Safety analysis of DAA therapy

None of the patients discontinued treatment because of adverse events. No death was observed during study period. Serious adverse events were observed in 3 patients and included arteritis of the leg vessels, which resolved after the end of treatment, asymptomatic QT prolongation, and gastrointestinal hemorrhage (**Table 2**). Hemoglobin level was stable except in 1 patient who developed gastrointestinal hemorrhage. Among a few patients who developed articular bleeding episodes during the study period, all patients had some bleeding episodes before the initiation of DAA therapy.

**Table 2:** Adverse events observed during the study period*.

Events	n	% (95% confidence interval)
Discontinuation of treatment due to adverse event	0	
Death	0	
Any adverse events	18	66 (46.0–83.5)
Grade 3 or 4 adverse events	3	11 (2.4–29.2)
Arteritis affecting leg vessels	1	
QT prolongation	1	
Gastrointestinal hemorrhage	1	
Common adverse events†	12	44.4 (25.5–74.5)
Anorexia	3	
Constipation	2	
Cough	2	
Headache	2	
Rash	2	
Malaise	1	
Laboratory abnormalities	2	0.07 (0.01–0.24)
Anemia	1	
HIV failure	1	

*HIV: human immunodeficiency virus.

†Listed are all adverse events that appeared after initiation of DAA therapy.

### Safety and efficacy of cART during DAA therapy

No side effects caused by cART were observed during DAA therapy, and none of the patients showed a decrease in CD4+ cell count. However, one patient acquired HIV drug resistance against raltegravir during treatment with ledipasvir and sofosbuvir (**Table 3**). Although this patient had had NRTI resistant mutations already before DAA therapy but had been treated successfully with abacavir plus tenofovir and raltegravir for 8 years (#2 patient in **S1 Table**) and patients’ compliance for treatment was good during study period, resistant mutations against raltegravir was new, acquired at 8 weeks after initiation of DAA therapy (**Table 4**).

**Table 3:** Clinical course of the patient who acquired new resistance mutations during DAA therapy*.

Week	-24	-12	0	4	8	12	24	30	33	36
DAA	none	none	L/S	L/S	L/S	L/S	none	none	none	none
cART	†	†	†	†	†	†	†	‡	‡	‡
HCV-RNA (log IU/mL)	5.2	N/A	5.6	TND	TND	TND	TND	TND	TND	TND
AST U/L	34	51	39	23	25	30	27	28	18	17
ALT U/L	33	72	42	22	26	31	27	25	15	12
HIV-RNA (copies/mL)	<20	<20	<20	<20	56	110	410	1400	<20	<20
CD4+ count (cells/μL)	215	227	260	220	248	334	263	237	N/A	342

The day of initiation of DAA therapy was set as “week 0”. DAA therapy was continued till week 12. cART was switched on week 30.

*cART: combination antiretroviral therapy, DAA: direct acting antivirals, L/S: ledipasivir plus sofosbuvir

†tenofovir plus abacavir plus raltegravir

‡darunavir plus ritonavir plus dorutegravir, N/A: not assessed, TND: target not detected, HCV: hepatitis C virus, HIV: human immunodeficiency virus.

**Table 4:** HIV-1 drug resistance mutations in a patient who acquired new resistance mutations during DAA therapy*.

Resistance testing in 2008†	
Reverse Transcriptase	41L, 67N, 69N/D, 70R, 184V, 215F, 219Q
Protease Inhibitor	10V, 13V, 20M/K, 33V, 36M/V, 46M/I, 62V, 64V, 74A/T, 88D/N, 90M/L
Integrase Inhibitor	Not tested
Resistance testing in 2016 (only newly acquired resistance mutations are listed)‡	
Reverse Transcriptase	179I, (184V disappeared)
Protease Inhibitor	30N
Integrase Inhibitor	140S, 148R

*This patient is the same as Patient #2 in S1 Table.

†Drug resistance testing was performed in 2008 (8 years before DAA therapy). Resistance mutation against integrase inhibitor was not tested at that time because integrase inhibitor was not administered in this patient until 2008. This patient had been treated previously with zidovudine, didanosine, stavudine, lamivudine, abacavir, tenofovir, saquinavir, nelfinavir, and ritonavir-boosted lopinavir. He had been treated with abacavir plus tenofovir and ritonavir boosted lopinavir from 2002 to 2008. Due to drug-induced liver injury, cART was switched to abacavir plus tenofovir and raltegravir in 2008. This was associated with suppression of HIV-1 viral load to undetectable level for 8 years until the DAA therapy in 2016.

‡Newly acquired resistance was tested at 18 weeks after the end of DAA therapy in 2016. DAA: direct acting antivirals, cART: combination antiretroviral therapy.

**Fig 2:**
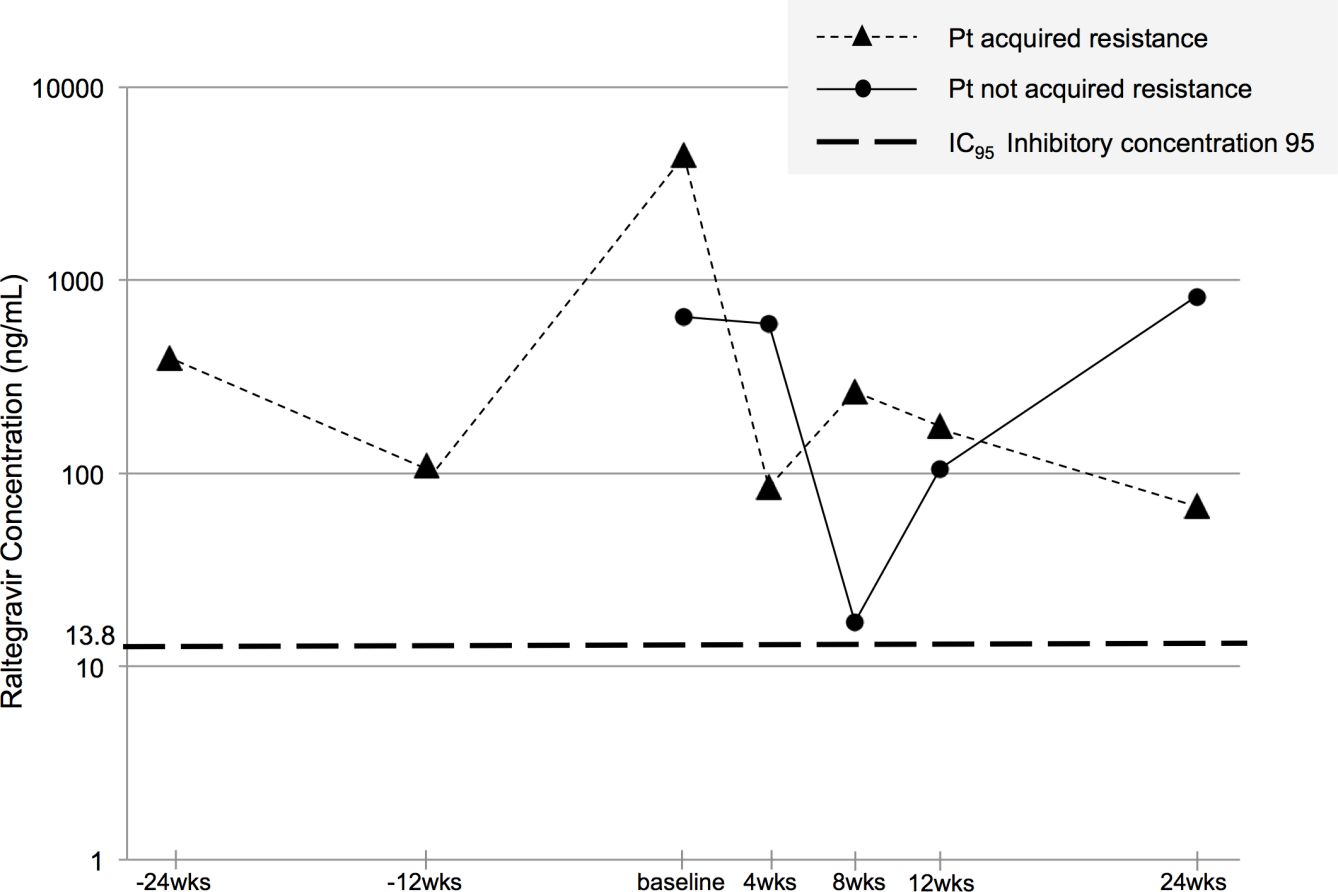
Plasma trough concentrations of raltegravir before, during, and after DAA therapy. Ordinate: logarithmic data. Raltegravir trough concentrations were assessed in 2 out of 7 patients who were on raltegravir during DAA therapy. Pt: patient, IC_95_: inhibitory concentration 95, DAA: direct acting antivirals.

We assessed plasma trough concentration of raltegravir in 2 out of 7 patients who were on raltegravir during DAA therapy and whose blood samples were available for analysis of the trough levels including the patient who acquired raltegravir resistance (**Fig 2**). In both cases, the trough concentrations did not fall below the inhibitory concentration 95 (IC_95_; >14 ng/mL) during DAA therapy.

### Changes in markers of liver fibrosis and HCC

**Fig 3:**
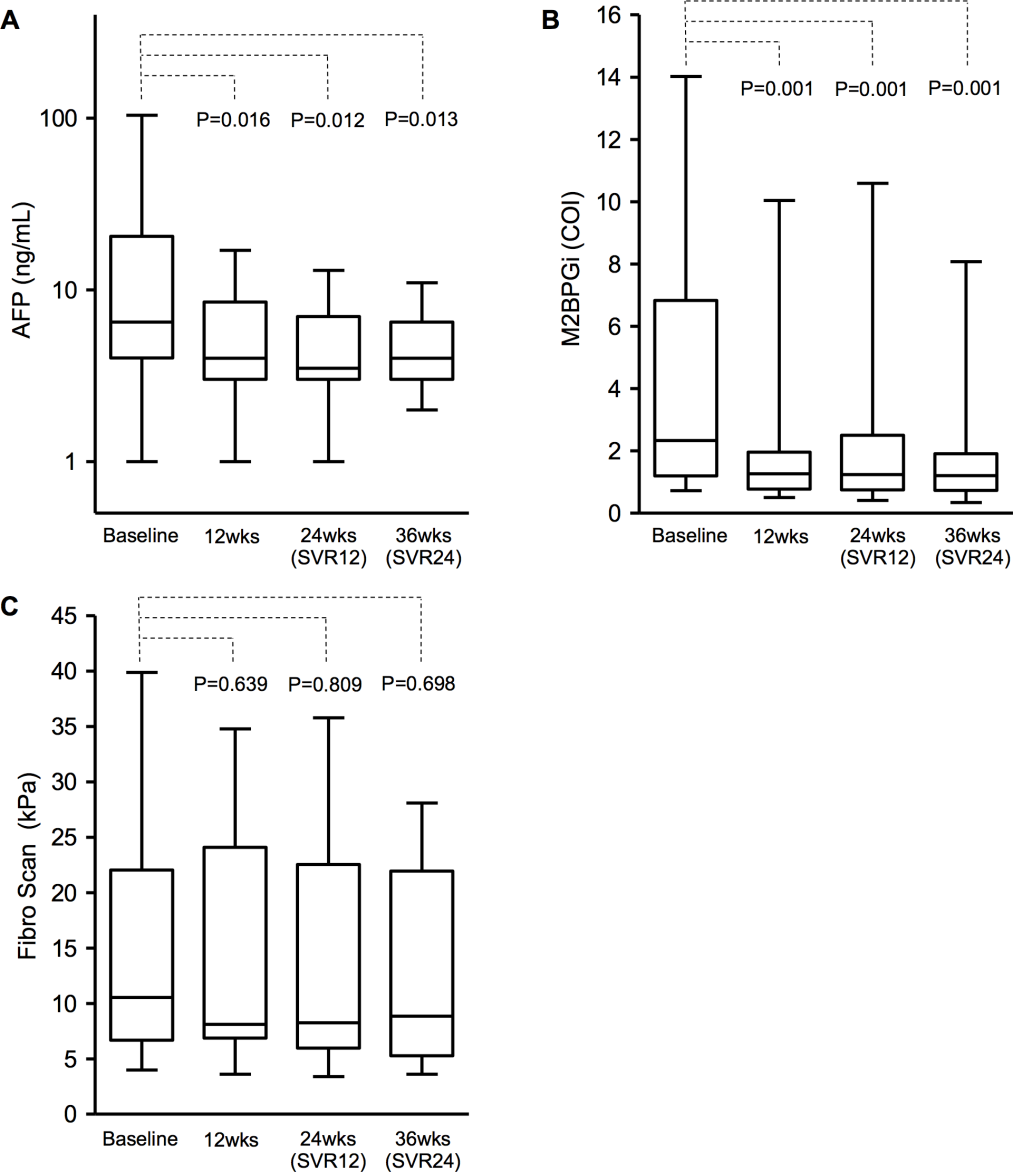
Changes in markers of liver fibrosis and HCC during and after DAA therapy. Box plots of the indicated parameters. (A) Logarithmic value of AFP α-fetoprotein. The datum of one patient with extremely high AFP value (1399 ng/mL) does not appear in the graph. (B) M2BPGi: mac-2 binding protein glycosylation isomer. COI: cutoff index. (C) Fibro Scan score represents the median of 10 procedures. *P*-values are for comparison of data of baseline and 12 weeks, baseline and 24 weeks, and baseline and 36 weeks, by the paired t-test. SVR: sustained virologic response.

In most patients, AFP (**Fig 3A**), M2BPGi (**Fig 3B**), and FS scores (**Fig 3C**) decreased during and after DAA therapy. In one patient, AFP was extremely high at the time of initiation of therapy (1399 ng/mL) but decreased to the normal level after DAA therapy. This patient was an outlier and excluded from statistical analysis. Changes in AFP and M2BPGi between baseline and each assessment point were statistically significant. At week 36, 6 patients had high M2BPGi score and also high FS score (>15.0 kPa). Among them, 2 patients had high AFP values (>10 ng/mL) and also high M2BPGi (3.07 and 6.6 cutoff index) and FS scores (21.0 and 21.3 kPa) at week 36. For the above 6 patients, the median age was 46, all had cirrhosis at baseline, 4 were HCV GT 1, 1 was GT 3, and 1 was GT4. None of the patients developed HCC during and after DAA therapy by week 36, as confirmed by ultrasonography and enhanced CT.

## Discussion

Among the HIV/HCV coinfected Japanese patients with inherited bleeding disorders who received interferon-free DAA therapy, all patients achieved sustained virologic response (SVR12 and 24) regardless of past anti-HCV treatment histories with interferon or the presence of compensated cirrhosis. The high treatment efficacy rate of DAA therapy for patients with inherited bleeding disorders reported here is similar to that documented in previous studies [10, 11].

Serious adverse events were observed in 3 patients. In one patient who was treated with ledipasvir and sofosbuvir for GT1 HCV, arteritis of the leg vessels was observed during DAA therapy but resolved soon after the end of treatment with initiation of antiplatelet drug therapy combined with calcium antagonist. However, the association between arteritis and DAA was not clear in this patient. In one patient who was treated with ledipasvir and sofosbuvir for GT1 HCV, grade 3 QT prolongation was observed at 4 weeks after the initiation of DAA therapy. Because the patient did not have any symptoms and the degree of QT prolongation remained stable without any further deterioration, we continued DAA therapy and QT prolongation disappeared spontaneously at 10 weeks after the initiation of DAA therapy without any medications. In this regard, one previous study also reported QT prolongation in a single patient with heart disease [19]. Interestingly, however, our patient did not have history of heart disease. The association between arteritis and DAA was not clear in this patient. The other patient who was treated with ledipasvir and sofosbuvir for GT1 HCV developed a single gastrointestinal bleeding episode during DAA therapy but the bleeding resolved after administration of blood coagulation product and digestive tract rest for several days. This patient had the same complication several times at 4 months before the initiation of DAA therapy and until more than 1 year after the end of DAA therapy. The bleeding was considered to be due to disorder of the mucus membrane of the small intestine associated with the inherited bleeding disorder, rather than being related to DAA therapy.

Among the patients who had been treated previously with antiretrovirals (ARVs) in the past, one patient acquired drug resistance against raltegravir, with resultant failure of HIV therapy during DAA therapy. A previous study involving healthy subjects reported minimal changes in the pharmacokinetics of antiretroviral drugs and sofosbuvir; raltegravir AUC was reduced by 17% by sofosbuvir [20]. The efficacy of raltegravir seems to depend on intracellular binding of the drug to the preintegration complex as well as on critical plasma concentrations [21]. However, it is known that intracellular raltegravir concentrations are lower than plasma raltegravir concentrations. Unfortunately, there are no data on intracellular raltegravir concentrations when it is administered with ledipasvir and sofosbuvir. It is possible that yet unknown intracellular drug-drug interactions (e.g., p-glycoprotein, which is the same efflux transporter of raltegravir, ledipasvir and sofosbuvir [22–25]) contribute to changes in the pharmacokinetics of intracellular raltegravir. Our patient had had multiple NRTI resistance mutations already. Therefore, it is possible that a slight decrease in intracellular raltegravir concentration could cause treatment failure. In a clinical study of 1196 patients who were treated with DAA for HCV, 26.9% were treated with raltegravir for HIV and none showed clinically significant interaction [26]. In another study, 17 patients were switched to raltegravir, tenofovir and emtricitabine following the initiation of HCV therapy, and none showed HIV virologic failure [27]. Although interactions among ledipasvir or sofosbuvir and raltegravir are considered minimal and clinically insignificant [26], patients coinfected with HIV and who have history of previous drug resistance should be monitored carefully for any new drug resistance against any ARVs.

Male sex, older age, high FS score and high AFP value are known independent predictors of HCC [28–31]. Most patients with inherited bleeding disorders were males. Although they were relatively young (median age: 45, IQR 43–48), they had more than 30-year histories of HCV infection and were therefore considered at high risk of HCC. M2BPGi, a well-known marker of fibrosis, is also considered a useful predictor of HCC [32, 33]. Although the preventative effect of interferon-based therapy against HCC is known, some reports showed progression of liver fibrosis to HCC development after eradication of HCV during the interferon era [13, 14]. Whether DAA therapy provides similar anticancer effect remains to be studied. In our study, although AFP and M2BPGi returned to normal levels in most patients, their levels remained high in 2 and 6 patients, respectively, at week 36. These two patients are considered to be at high risk for HCC and their liver status should be assessed carefully regardless eradication of HCV.

Our study is limited by the small sample size and short observation period. Since the number of patients with inherited bleeding disorders is relatively small, we need further data on DAA therapy of these patients. A longer observational period is necessary to explore the anti-HCC effect of DAA therapy.

## Conclusions

DAA therapy was effective for HCV infection. However, we should monitor adverse effects carefully in HIV-1 co-infected Japanese patients with inherited bleeding disorders. Furthermore, one should consider background drug resistance mutations against ARVs because such patients often have history of aggressive treatment with ARVs and treatment failures.

## Supporting information

S1 Checklist Trend checklist. TREND statetment checklist.
(PDF)

S1 Table Supplementary Table. Current cART, past ARVs, and number of cART switch.
(DOCX)

S1 Protocol Protocol English. Study protocol for the off-label use in English.
(DOCX)

S2 Protocol Protocol Japanese. Study protocol for the off-label use in Japanese.
(DOCX)
