# Supplementary material for: Interferon-free therapy with direct acting antivirals for HCV/HIV-1 co-infected Japanese patients with inherited bleeding disorders
Source: PLoS One. 2017 Oct 18;12(10):e0186255. doi: 10.1371/journal.pone.0186255 (PMC5646795; doi:10.1371/journal.pone.0186255)
Supplement: S1 Table — Current cART, past ARVs, and number of cART switch. (DOCX) [file pone.0186255.s002.docx]

| **Pt #** | **Current cART†** | **past ARVs used‡** | **cART switch#§** |
| --- | --- | --- | --- |
| 1¶ | 3TC/ABC+ETR+RAL | d4T, 3TC, ABC, TDF, EFV, RTV, NFV, APV, LPV, DTG | 6 |
| 2¶ | ABC+TDF+RAL | AZT, ddI, d4T, 3TC, ABC, TDF, EFV, RTV, SQV, NFV, LPV | 5 |
| 3¶ | FTC/TDF+RAL | AZT, d4T, ddC, 3TC, ABC, FTC, TDF, EFV, SQV, IDV | 4 |
| 4¶ | FTC/TD+ETR+RAL | AZT, ddI, d4T, 3TC, FTC, TDF, NVP, EFV, SQV, RTV, IDV, NFV, LPV | 15 |
| 5¶ | 3TC /ABC+ETR+RAL | AZT, ddI, d4T, ddC, 3TC, ABC, FTC, TDF, EFV, RTV, APV, LPV | 5 |
| 6¶ | DRV/r+RAL | ddI, d4T, 3TC, FTC, TDF, EFV, NFV, DRV | 5 |
| 7¶ | FTC/TDF+RAL | None | 0 |
| 8 | FTC/TDF+ETR | AZT, ddI, d4T, ddC, 3TC, ABC, FTC, TDF, NVP, EFV, SQV, RTV, NFV, LPV, RAL, | 20 |
| 9 | DRV/r+MVC | AZT, ddI, 3TC, ABC, FTC, TDF, NVP, EFV, ETR, SQV, RTV, ATV, DRV, RAL | 12 |
| 10 | 3TC/ABC+NVP | ddI, d4T, 3TC, ABC, TDF, NVP, EFV, RPV | 5 |
| 11 | FTC/TDF+DTG | AZT, ddI, d4T, ddC, 3TC, ABC, FTC, TDF, NVP, EFV, RTV, SQV, NFV, LPV, RAL | 15 |
| 12 | FTC/TDF+DTG | AZT, ddI, ddC, FTC. TDF. ATV, RAL | 6 |
| 13 | 3TC/ABC/DTG | AZT, d4T, 3TC, ABC, FTC, TDF, RTV, ATV, FPV, DRV, RAL, EVG, COBI | 14 |
| 14 | FTC/TDF+DTG | AZT, ddI, RTV, FTC, TDF | 3 |
| 15 | FTC/TDF+DTG | AZT, ddI, ddC, 3TC, ABC, FTC, TDF, NVP, EFV, SQV, RTV, IDV, NFV, APV, LPV, DRV, RAL | 14 |
| 16 | 3TC/ABC/DTG | AZT, ddC, 3TC, ABC, NVP, EFV, NFV, APV | 8 |
| 17 | LPV/r+DTG | AZT, d4T, 3TC, FTC, TDF, EFV, RTV, NFV, LPV | 5 |
| 18 | DRV/r+DTG | AZT, ddI, ddC, 3TC, ABC, TDF, EFV, RPV, SQV, RTV, NFV, APV, LPV, DRV, RAL, MVC, T-20 | 21 |
| 19 | FTC/TDF+DTG | AZT, 3TC, FTC, TDF, RTV, FPV | 3 |
| 20 | 3TC/ABC/DTG | AZT, ddI, d4T, ddC, 3TC, ABC, RTV, NFV, ATV | 5 |
| 21 | RPV+DTG | AZT, ddI, d4T, 3TC, NVP, EFV, RTV, NFV, APV, LPV, DRV, RAL | 21 |
| 22 | 3TC/ABC/DTG | AZT, ddI, d4T, RTV, NFV, LPV, RAL | 4 |
| 23 | FTC/TDF+DRV/r+DTG | AZT, ddI, d4T, ddC, 3TC, FTC, TDF, SQV, RTV, NFV, APV, LPV, DRV, RAL, T-20 | 17 |
| 24 | 3TC/ABC+DRV/r | AZT, ddI, d4T, 3TC, ABC, NVP, EFV, RTV, NFV, APV | 11 |
| 25 | FTC/TDF+RPV | d4T, 3TC, FTC, TDF, EFV, RTV, LPV, FPV | 4 |
| 26 | RPV+DTG | AZT, ddI, d4T, ddC, 3TC, ABC, FTC, TDF, EFV, ETR, RTV, IDV, NFV, DRV | 12 |
| 27 | FTC/TDF+DTG | AZT, ddI, d4T, 3TC, FTC, TDF, NVP, ETR, RPV, SQV, RTV, NFV, LPV, ATV, DRV, RAL, T-20 | 16 |
| †ARV regimens used during DAA therapy. ‡ARV regimen that had been used before current cART. §cART switch was due to HIV treatment failure or emergence of side effects. ¶Patients who were treated with RAL during DAA therapy.  cART: combination-active antiretroviral therapy, ARV: antiretroviral, DAA: direct acting antivirals, AZT: zidovudine, ddI: didanosine, d4T: stavudine, ddC: zalcitabine, 3TC: lamivudine, ABC: abacavir, FTC: emtricitabine, TDF: tenofovir, NVP: nevirapine, EFV: efavirenz, ETR: etravirine, RPV: rilpivirine, SQV: saquinavir, RTV: ritonavir, IDV: indinavir, NFV: nelfinavir, APV: amprenavir, LPV: lopinavir, ATV: atazanavir, FPV: fosamprenavir, DRV: darunavir, RAL: raltegravir, DTG: dolutegravir, EVG: elvitegravir, MVC: maraviroc, T-20: enfuvirtide, COBI: cobicistat. | | | |
