# Supplementary material for: Interferon-free therapy with direct acting antivirals for HCV/HIV-1 co-infected Japanese patients with inherited bleeding disorders
Source: PLoS One. 2017 Oct 18;12(10):e0186255. doi: 10.1371/journal.pone.0186255 (PMC5646795; doi:10.1371/journal.pone.0186255)
Supplement: S1 Protocol — Study protocol for the off-label use in English. (DOCX) [file pone.0186255.s003.docx]

**Pilot study to evaluate the efficacy and safety of the combination of sofosbuvir and daclatasvir for HCV genotype 3 infection in HIV and HCV co-infected hemophiliacs**

Protocol version 1.1

UMIN CTR ID: 000019659

Research representative:

Shinichi Oka, MD, PhD

AIDS Clinical Center, National Center for Global Health and Medicine

162-8655　1-21-1 Toyama, Shinjuku, Tokyo, Japan

TEL: 03-3202-7181

Fax: 03-5273-5193

e-mail: oka@acc.ncgm.go.jp

[Version record]

- August 27, 2015. Ethical Committee, National Center for Global Health and Medicine (version 0.1)
- September 25, 2015. Ethical Committee, National Center for Global Health and Medicine (version 1.1)
- October 20, 2015. Ethical Committee, National Center for Global Health and Medicine (version 1.1)

**1. Background**

It is estimated that one and a half to two million Japanese are infected with hepatitis C virus (HCV). Although there is about 30% chance of spontaneous resolution of HCV infection in mono-infected patients, this chance is only 10% in patients co-infected with human immunodeficiency virus (HIV) ^1)^. In chronic HCV infection, the rate of spontaneous resolution of infection is only 0.2% per year and continuous inflammation causes liver fibrosis, which could potentially progress to cirrhosis or hepatocellular carcinoma (HCC) ^2)^. HIV-HCV co-infected HCC patients are younger and more frequently symptomatic than HIV-negative HCV patients^3)^.

More than 30 years ago, several Japanese hemophiliacs were co-infected with HCV and HIV-1, after they received contaminated blood products. Liver cirrhosis has already developed in some of these patients since HCV infection in 1980’s. In the era of combination-active antiretroviral therapy (cART), the main cause of death in this population is not related to HIV-1 but rather to HCV-related diseases.

The efficacy of dual therapy of interferon (IFN) and ribavirin in HCV is about 40 to 50%. In 2014, the Japanese medicare system subsidized IFN-free treatment with the combination of asunaprevir and daclatasvir for patients with chronic hepatitis caused by HCV genotype (GT)-1. Moreover, the combinations of sofosbuvir-ribavirin for GT2 HCV infection and ledipasvir-sofosbuvir for GT1 HCV infection were approved in 2015. On the other hand, treatment for HCV GT3 has not been approved yet in Japan. This is mainly due to the low incidence of GT3 infection in Japan especially in non-hemophiliacs. Yotsuyanagi et al. reported that among 226 hemophiliacs in 17 institutions, 13.3% of patients had HCV GT3 infection, and that 12 of 122 patients required treatment for HCV GT3 infection ^3)^. In our hospital, 4 of 37 HIV/HCV co-infected patients required treatment for HCV GT3. Three out of 4 had cirrhosis with Child-Pugh score 4 and required early initiation of treatment.

Twelve-week treatment with sofosbuvir and daclatasvir was reported in two clinical studies to be highly efficient (14 out of all 14; 100%, and 10 out of 10 patients; 100%) ^4, 5)^. None of the enrolled patients discontinued the treatment, and the outcome was considered better than treatment with interferon and ribavirin ^4, 5)^. However, the combination of sofosbuvir and daclatasvir has not been approved yet in Japan.

The aim of this pilot study is to evaluate the effectiveness and safety of treatment with daclatasvir and sofosbuvir for HCV GT3 in HIV/HCV co-infected patients. Since this treatment has been used in only a few patients, we anticipate that this pilot study will provide important clinical information. Moreover, the results of this study will contribute to the planning of clinical trials for the treatment of HCV genotype 3 infection in Japan.

**2. Objective**

The main objective of this trial is to evaluate the efficacy and safety of dual therapy of sofosbuvir and daclatasvir for HCV genotype 3 infection in HIV/HCV co-infected hemophiliacs.

**3. Subject selection**

Subjects must meet all of the following inclusion criteria, and subjects who meet any of exclusion criteria listed below are excluded from the study.

*Inclusion criteria:* i) hemophiliacs infected with HIV-1 and HCV GT3, ii) patients who failed to respond to IFN-based therapy for HCV, iii) subjects positive for HCV-RNA at the time of enrollment, iv) patients on antiretroviral treatment for more than 8 weeks and have evidence of HIV-1 viral suppression (HIV-1 RNA <200 copies/mL) with a CD4+ count of >100 cells/μL at the time of enrollment, v) patient must be willing to avoid becoming pregnant from the initiation of direct-acting antiviral (DAA) therapy to 14 weeks after the end of DAA therapy (total 26 weeks), vi) patient must be >20 years old, and vii) consent for administration of drugs with disapproval usage.

Exclusion criteria: i) patients with non-compensated cirrhosis, ii) patients with severe renal impairment (eGFR <30 ml/minute/1.73 m^2^), iii) patients positive for HBs antigen, iv) patients with active opportunistic infection, v) patients on medications that are contraindicated for concomitant use with sofosbuvir and daclatasvir (e.g., rifampicin, rifabutin, phenytoin, carbamazepine, phenobarbital, dexamethasone, St John's wort), and v) patients who are judged inappropriate to participate in the study for any other reason by the investigator.

**4. Number of subjects and study duration**

4.1. Planned number of subjects: 4

4.2. Study duration:

4.2.1. Enrollment period: From the day of approval by ethics committee to March, 2017

4.2.2. Study period: After the day of enrollment to March, 2017

**5. Therapeutic regimen**

5.1. Study drugs

Patients receive sofosbuvir 400 mg plus daclatasvir 600 mg once daily for 12 weeks.

*Dose adjustment*

- Patients on antiretroviral therapy with protease inhibitor plus booster, the dose of daclatasvir will be adjusted to 30 mg.
- Patients on antiretroviral therapy with non-nucleic acid reverse transcriptase inhibitor, excluding rilpivirine, the dose of daclatasvir will be adjusted to 90 mg.

5.2. Study schedule

- The investigator plans to continue the treatment for 1 year after the day of enrollment
- The day of initiation of therapy is defined as “Day 1”.

5.3. Tests schedule

In principle, clinical examinations and laboratory tests will be conducted at 2, 4, 8, 12, and 24 weeks after the day of initiation of treatment.

**6. Clinical examination and follow-up**

6.1. Baseline assessments

These included age at first consultation, sex, classification of inherited bleeding disorders, HCV treatment history infection, presence of cirrhosis, HBs antigen, presence of alcoholic liver injury, presence of fatty liver, past medical history, other complications, and past medical history.

6.2. Clinical parameters

These include body weight, body temperature, blood pressure, clinical symptoms and signs, physical examination, blood count, biochemistry [serum albumin, aspartate aminotransferase (AST), alanine aminotransferase (ALT), total bilirubin (T. Bill), gamma glutamyl transpeptidase (γ-GTP), creatinine (Cre), estimated glomerular filtration rate (eGFR), blood urea nitrogen (BUN), lipase), urinalysis, HCV-RNA load, prothrombin (PT) activity, prothrombin time-international normalized ratio (PT-INR), HIV-RNA load, CD4+ count, electrocardiogram (ECG), history of antiretroviral therapy, and other concomitantly used medications.

**7. Collection of biological samples**

Blood samples will be collected on the day of the initiation of therapy and 2, 4, 8, 12, and 24 weeks after the initiation of therapy. After centrifugation, the samples are preserved in deep freezer at -80°C in the laboratory of AIDS clinical center, National Center for Global Health and Medicine. Samples are managed with study ID. When these samples are used after the completion of this study, consent in writing has to be obtained again.

**8. Criteria for discontinuation of drugs**

The investigator must consider discontinuation of the administered drugs when one of the following event is documented:

- QTc >500 msec (ECG)
- 2^nd^ and 3^rd^ degree atrioventricular block (ECG)
- Development to non-compensated cirrhosis
- ALT >5 times the baseline value or 10 times the upper limit of normal
- Total bilirubin >5 times the baseline value or 10 times the upper limit of normal
- PT-INR more than twice the upper limit of normal
- Platelet count <25,000/mL
- Grade 4 laboratory abnormality suspected to be linked to the study drugs
- Consent withdrawal from the subject
- Continuation of study drug is considered clinically harmful to the subject
- Any reason that favors discontinuation of the study drug (e.g. dysphagia, change of hospital)

**9. Evaluation**

9.1. Evaluation of efficacy

Sustained virological response: Successful treatment is defined by sustained virological response at 12 weeks after the end of treatment. Viral failure is defined as breakthrough, recurrence, or detectable viral load for any other reason.

**10. Drug information**

10.1. Sofosbuvir (Generic name: Sobarudi®)

Sofosbuvir is a nucleotide analog and a potent and selective inhibitor of NS5B-directed HCV replication. It is efficacious *in vitro* against HCV GT 1a, 1b, 2a, 2b, 3a, 4a, 5a, and 6a. Treatment with the combination of sofosbuvir and ribavirin for GT2 HCV infection was approved in Japan in March 2015. However, sofosbuvir is contraindicated in patients with severe renal impairment (eGFR <30 mL/minute/1.73 m^2^ and patients on hemodialysis.

Rifampicin, carbamazepine, phenytoin, and St John’s wort should not be given to patients treated with the combination of daclatasvir and sofosbuvir. Care should be taken when prescribing rifabutin and phenobarbital for patients treated with the combination of daclatasvir and sofosbuvir

*Adverse effects:* In one Japanese phase III clinical trial, 73% of patients had at least one treatment-related adverse event. Among these patients, 84% had mild (grade 1) events. The most common adverse events were nasopharyngitis in 29% of the patients, anemia in 12%, headache in 10%, fatigue in 7%, and rash in 6%. No grade 4 adverse events were observed in this series. Two cases of treatment-related grade 3 adverse events were observed (anemia and hyperbilirubinemia). None of the patients discontinued treatment based on treatment-related adverse events.

10.2. Daclatasvir (Generic name: Daklinza®)

Daclatasvir is an inhibitor of NS5A-directed HCV replication.

Rifampicin, rifabutin, carbamazepine, phenytoin, phenobarbital, dexamethasone, and St John’s wort should not be administered to patients treated with the combination of daclatasvir and sofosbuvir. Care should be taken when prescribing azole, protease inhibitor of HIV, cobicistat, clarithromycin, telaprevir, efavirenz, digoxin, rosuvastatin, atorvastatin, fluvastatin, simvastatin, pitavastatin, and pravastatin for patients treated with the combination of daclatasvir and sofosbuvir

*Adverse events:* In a Japanese phase III clinical trial of daclatasvir and asunaprevir, nasopharyngitis and headache were the two most common adverse events recorded (29%, each), followed by anemia 12%, headache 10%, fatigue 7%, and skin rashes 6%. No grade 4 adverse events were observed in this series. Grade 3 adverse events (high ALT and AST levels) were observed in 16 (7.2%) and 2 (4%) patients, respectively. Ten cases (4.5%) discontinued treatment during the study period.

10.3. The combination of sofosbuvir and daclatasvir

In a phase III clinical trial of sofosbuvir and daclatasvir (203 cases), the most common adverse events were fatigue in 34 (17%) patients, nausea in 26 (13%), headache in 23 (11%), diarrhea in 15 (7%), vomiting in 10 (5%), skin rashes in 9 (4%), insomnia in 8 (4%), stomachache in 7 (3%), cough in 7 (3%), vertigo in 6 (3%), and constipation in 6 (3%). None of the cases discontinued treatment based on treatment-related adverse events. No grade 4 adverse events were observed in that study. Two cases of treatment-related grade 3 adverse events were observed (anemia and hyperbilirubinemia). None of the patients discontinued treatment due to treatment-related adverse events. The following grade 3/4 abnormalities were observed: hyperbilirubinemia in 8 (4%) patients, transient increase in serum lipase in 7 (3%), increase in INR in 2 (1%), and increase in AST in 1 (<1%) patient.

**11. Management of adverse events**

At the time of onset of adverse events, participants will receive proper medication(s). The study drugs will be withdrawn when the case meets withdrawal criteria or withdrawal is judged appropriate.

**12. Report of adverse events**

Upon the appearance of grade 3/4 adverse events or discontinuation of the study drugs, the information will be shared among investigators on the case report form (CRF-6) and reported to the research representative. At the time of onset of the following adverse events, research representative have to prepare a document on “Report for severe adverse event or failure” and report to the general and ethics committee to the Clinical Research Promotion Office within 15 days. At the time of onset of unexpected adverse events, research representative have to publish the full details of the cases and report to the Minister for Health, Labour and Welfare.

List of severe adverse events:

- Death
- Life threatening events
- Hospitalization is required for treatment
- Onset of lifelong/significant disabilities/dysfunction
- Congenital abnormalities in offsprings
- Other adverse events graded as severe

**13. Standard therapy and basis of scientific rationality for research**

There is no standard therapy for HCV genotype 3 infection in Japan. In comparison, several studies have reported the efficacy and safety of treatment with sofosbuvir and daclatasvir for HCV genotype 3 infection in western countries^4)^.

**14. Advantages and disadvantages of participation in this study**

14.1. Expected advantages

Daclatasvir is approved for use with asunaprevir for HCV genotype 1 infection, and sofosbuvir is approved for use with ledipasvir for HCV genotype 1 infection and with ribavirin for HCV genotype 2 infection. Moreover, the efficacy and safety of treatment with daclatasvir and sofosbuvir for HCV genotype 3 infection is well documented in European and North American countries. Since the participants in this study have not been treated previously with interferon for HCV genotype 3 infection, participation in this study will probably reduce the risk of development of cirrhosis and hepatocellular carcinoma.

14.2. Expected disadvantages

To evaluate the safety of the study drugs, the participants are required to visit the hospital several times, provide several blood samples, and undergo repeated ECG recordings. Furthermore, some can potentially develop unexpected adverse events. In case of the appearance of the latter, the participants will need to visit the hospital more frequently and receive proper treatment.

**15. Ethical issues**

15.1. Protection of privacy of participating subjects

The investigators and all other project personnel are responsible for the conduct of this research protocol in accordance with the ethics principals outlined in the Declaration of Helsinki and Ethical Guidelines for Medical and Health Research Involving Human Subjects published by Ministry of Health, Labour, and Welfare, Japan.

15.2. Informed consent

The investigator is responsible for obtaining written informed consent from each individual participating in this study. The investigator should provide adequate explanation of the study to the participating subjects, together with a copy of the document approved by the ethic committee. The investigator should explain to each individual that refusal to participate and withdrawal from the study will not affect the quality of clinical care. Each informed consent will be appropriately signed by the participating subject and kept in the patient medical record.

15.3. Procedure to receive informed consent from legal representative

Subjects of this study are at extremely high risk of development of cirrhosis and hepatocellular carcinoma should the prescribed medications fail to eradicate HCV. Therefore, special consideration will be given to subjects who are not able to provide informed consent. Adult subjects who are not able to give informed consent will be assessed by more than two non-investigators and then the investigator will receive informed consent from a legal representative. In this study, legal representatives are limited to the parents of the subject. Investigator will explain this study in plain terms and provide printed documents related to the study. The Investigator will leave a record of the discussion on participation in this study, including the circumstances of receiving informed consent, in the medical record.

15.4. Confidentiality

Upon entry into the study, the subject will be anonymized by using a research ID. This will be linked to the medical record number. The anonymized data list will be handed to the planning director-general.

**16. Preservation and disposition of biological samples and information**

Individual information and data collected in this study will be preserved for 5 years after the study completion or 3 years after the last publication of the study outcome. All study-related data and information will be destroyed physically upon study disposal. The digital data stored on digital media will be deleted and wiped out. Because the collected blood (EDTA plasma and serum) is valuable material, the samples will be preserved after the study until consent is withdrawn. The blood samples will be disposed off in the same way as the other individual information and data.

**17. Report to the director of the research institution**

The investigator will report the following events to the director through the Clinical Research Promotion Office. Progress of the study will be reported regularly at the end of August of each year.

- - Any severe adverse event
  - Discontinuation of the study
  - Restart of the study

**18. Financial support for the study and conflicts of interest**

The study will be funded by a grant from the National Center for Global Health and Medicine. There is no conflict of interest in this study.

**19. Information disclosure**

The research outline will be registered with the UMIN-CTR for public disclosure of information.

**20. Cost burden and reward of participants**

The study drugs and all clinical and laboratory tests will be paid for from the research funds. There is no additional payment by participants. No financial reward will be paid to participants.

**21. Compensation for any health damage by this study**

There will be no financial compensation for any health damage caused by participation in this study by the Investigator. Any such compensation will be made by the public liability insurance that covers the attending physician. Regardless of any such compensation, the investigator is not liable and will practice medicine appropriately.

**22. Monitoring and auditing**

In principle, all participants will be monitored for one month after enrollment in the study for clinical assessment and safety. All participants will be also monitored at the end of treatment. Monitoring will be achieved according to the list described in 22.1 below. The research representative will select the monitoring individual from the research assistance. The monitoring individual will report to the research representative. Any major severe violation and departure from the protocol will be reported to the chief director by the research representative. No auditing will be done in this study.

22.1. List of items to be monitored

- All documents related to the informed consent
- Eligibility of subject selection
- Any adverse events, including no such events
- Adherence to the protocol, or lack of such adherence
- All matters related to safety and study progress
- Collation of case reports and original data
- Status of reservation of subject files and study documents

**23. Research organization**

23.1. Research representative

Hiroyuki Gatanaga, MD, PhD. AIDS Clinical Center, National Center for Global Health and Medicine, 〒162-8655　1-21-1 Toyama, Shinjuku, Tokyo, Japan. TEL: 03-5273-5193. Fax: 03-5273-5193. e-mail: oka@acc.ncgm.go.jp

23.2. Research assistants

- Yoshimi Kikuchi, MD. AIDS Clinical Center, National Center for Global Health and Medicine
- Hiroyuki Gatanaga, MD, PhD, AIDS Clinical Center, National Center for Global Health and Medicine
- Masashi Mizokami, MD, Genome Medical Sciences, Research Institute, National Center for Global Health and Medicine
- Mikio Yanase, MD, Department of Gastroenterology, National Center for Global Health and Medicine
- Mikiko Ogata, MD, AIDS Clinical Center, National Center for Global Health and Medicine
- Misao Takano, MD, CRC, National Center for Global Health and Medicine

**23.3. Contact person**

Hiroyuki Gatanaga, MD, PhD. AIDS Clinical Center, National Center for Global Health and Medicine. 〒162-8655, 1-21-1 Toyama, Shinjuku, Tokyo, Japan. TEL: 03-5273-5193. e-mail: oka@acc.ncgm.go.jp

**24. References**

1. The Japan Society of Hepatology. Guideline for hepatitis C treatment (ver 3.5)

2. Vogel M et al. Acute hepatitis C infection in HIV-positive patients. *Curr Opin Infect Dis* 24:1-6, 2011.

3. Brau N et al. Presentation and outcome of hepatocellular carcinoma in HIV-infected patients: U.S.-Canadian multicenter study. *J Hepatol* 47:527-37, 2007.

4. Wyles DL et al. Daclatasvir plus Sofosbuvir for HCV in patients co-infected

with HIV-1. *N Engl J Med* 373:714-725, 2015.

5. Lacombe K et al. Daclatasvir plus Sofosbuvir with or without ribavirin in patients with HIV-HCV coinfection: Interim analysis of a French multicenter compassionate use program. A1444-258 Study IAS 2015 Jul 19-22, Vancouver
