# Supplementary material for: Interferon-free therapy with direct acting antivirals for HCV/HIV-1 co-infected Japanese patients with inherited bleeding disorders
Source: PLoS One. 2017 Oct 18;12(10):e0186255. doi: 10.1371/journal.pone.0186255 (PMC5646795; doi:10.1371/journal.pone.0186255)
Supplement: S2 Protocol — Study protocol for the off-label use in Japanese. (DOCX) [file pone.0186255.s004.docx]

血友病HIV/HCV重複感染患者でHCV遺伝子3型に対する

ソホスブビル＋ダクラタスビルの有効性・安全性を検証するパイロット試験

実施計画書　Version　1.1

UMIN CTR ID: 000019659

研究代表者　岡 慎一

国立国際医療研究センターエイズ治療・研究開発センター

〒162-8655　東京都新宿区戸山1-21-1

TEL：03-5273-5193

FAX：03-5273-5193

e-mail：oka@acc.ncgm.go.jp

2015年8月27日 国立国際医療研究センター倫理委員会提出（Version 1.0）

2015年9月25日 国立国際医療研究センター倫理委員会提出（Version 1.1）

2015年10月20日 国立国際医療研究センター倫理委員会承認（Version 1.1）

1. **研究の背景と意義**

本邦では、C型肝炎患者数は150万～200万人と推定されている。HCV感染が一旦成立すると、健康成人への感染であっても、感染例の約70%で慢性感染へと移行する。慢性化した場合、ウイルスの自然排除は年率0.2%とまれであり、HCV感染による炎症の持続により肝線維化が惹起され、肝硬変や肝細胞癌へと進展する^1)^。 HCVとHIVの重複感染患者では、HCVが慢性化する率は約90%と健常成人と比べさらに高い^2)^。 また重複感染患者はHCV単感染患者に比較して肝障害の進行が速いことが知られている^3)^。 本邦の血友病HIV感染患者の多くが血液製剤によりHIVと同時にHCVに感染している。これらの血友病HIV/HCV重複感染例 は、1980年代のHCV感染から長期間が経過し肝線維化が進行している例が多い。HIVがコントロールできるようになった現在、肝硬変/肝臓癌が主たる死亡原因となっており、むしろ血友病HIV/HCV重複感染患者にとって、C型肝炎の方がHIV感染症より大きな問題となっている。

C型肝炎の治療はインターフェロン(IFN)、リバビリンなどを組み合わせた治療が中心であったが、その治療効果は40～50%程度と低かった。しかし2014年にGenotype 1 (GT1）のC型肝炎に対してダクラタスビル＋アスナプレビルによるIFNを含まない抗HCV療法が可能となり、2015年にはソホスブビル＋リバビリンによりGenotype 2 (GT2) のC型肝炎に対して治療が承認された。また、ソホスブビル/レディパスビルの合剤 (ハーボニー)も認可され、ハーボニーによるGT1及びGT2のC型肝炎については保険適応の治療で高い治療効果が期待されている。

一方、Genotype 3 (GT3) のHCVによるC型肝炎については、これまで日本ではDAAによる保険で認可された治療法はない。これは、日本においては血友病以外のC型肝炎患者では、GT3がほとんどおらず、臨床試験が成立しないからである。しかし、東京大学の四柳らによる全国17施設の226名の血友病を対象とした研究では、GT3が13.3％存在し、治療を必要とする患者も122名中12名存在した。当院においては、治療を必要とする血友病HIV/HCV感染者37名中4名がGT3であり、今回のパイロット試験の対象者である。うち3例はChild-Pugh Aの肝硬変に進展しており早急な治療が必要とされており、救済医療的な側面からも迅速な対応が不可欠である。

海外の2つの臨床研究ではソホスブビルとダクラタスビルによる12週間の治療で、治療歴、および肝炎/肝硬変の進展にかかわらず、1つ目の研究では100％(10/10)、2つ目の研究でも100％（14/14）の高い治療効果が報告されている。この組み合わせによる治療は、治療の中断につながるような重篤な副作用を認めず、IFN＋RBVによる治療と比べて格段に優れていた^4)5)^。しかし、ソホスブビルとダクラタスビルは各々C型肝炎の治療薬としてすでに国内承認を受けているものの、この2剤による組み合わせの治療は、国内では保険診療の認可を受けていない。

本試験は、ソホスブビルとダクラタスビルをGT3のHCVによる血友病HIV/HCV重複感染患者に保険適応外で併用し、海外で報告されているように、有用かつ安全であることを検証するパイロット試験である。なお、海外でのGT3に対する症例数も少なく、本パイロット試験の意義は大きいと考えられる。また、この研究結果は、今後の日本における血友病HIV/HCVのGT3 によるC型肝炎に対する臨床試験を計画する上での参考資料となる。

1. **研究の目的**

血友病HIV/HCV重複感染者のうちGT3のHCVに感染した患者に対して、直接作用抗HCV薬ソホスブビル+ダクラタスビルを用いて治療し、その有効性、安全性を評価する。

1. **対象者**

以下の適格基準をすべて満たし、除外基準のいずれにも該当しない患者を対象とする。

- 1. 適格基準

・血友病HIV/HCV重複感染者でGT3のHCVに感染した患者

・過去にインターフェロンによる治療を失敗した症例または不適応だった患者

・登録時にHCV-RNA陽性

・登録時点で8週以上HIV-RNAが50 copies/mL未満、CD4陽性Tリンパ球が100/μL以

　上を維持している患者

・試験薬内服12週間および服薬終了後14週間（計26週間）、確実に避妊が出来る患者

・20歳以上

・各試験薬は保険適応になっているが、組み合わせについては未認可の薬剤の使用について

文書による同意が得られた患者

- 1. 除外基準

・非代償性肝硬変患者

・重度の腎機能障害（eGFR＜30ml/分/1.73m^2^）を有する患者

・HBs抗原陽性の患者

・治療が必要な活動性のある日和見感染症を合併している患者

・試験で使用される薬剤と併用禁忌になっている薬剤を使用している患者

　（リファンピシン、リファブチン、フェニトイン、カルバマゼピン、フェノバルビタール、

　　デキサメサゾン全身投与、セイヨウオトギリソウ含有食品）

・その他、担当医師が不適当と判断した患者

1. **症例数と研究期間**
   1. 予定症例数

4名

- 1. 研究実施期間

1. 登録期間　倫理委員会承認後～2017年3月
2. 研究期間　患者登録～2017年3月
3. **試験治療**
   1. 試験治療

ソホスブビル400mgとダクラタスビル60mg　1日1回、12週間内服

用量変更を必要とする場合

・ブースターを含むプロテアーゼ阻害薬でHIV治療をしている患者

ソホスブビル400mgとダクラタスビル30mg　1日1回、12週間内服

・リルピビリンを除く非核酸系逆転写酵素阻害剤でHIV治療している患者

ソホスブビル400mgとダクラタスビル90mg　1日1回、12週間内服

5.2 試験実施スケジュール

登録～治療開始まで：4週間以内

治療開始日をDay1として観察を開始する。

5.3検査スケジュール

原則として試験薬開始後2週、4週、8週、12週、24週に所定の観察と検査を実施する。

1. **観察項目**
   1. 背景因子

年齢、性別、血友病の分類、慢性C型肝炎の治療歴、肝硬変の有無、HBV感染の有無（HBsAg）アルコール性肝障害の有無、脂肪肝の有無、その他合併症、既往歴

- 1. 観察項目

体重、体温、血圧、自覚症状、他覚的身体所見、血算、生化学一般(*1)、尿一般検査、HCV-RNA量、PT活性％、INR、HIV-RNA量、CD4数、心電図所見、抗HIV薬、その他併用薬

(*1) Alb、AST、ALT、T.Bill、γGTP、血清Cre、eGFR、BUN、リパーゼ

1. **保存用検体の採取**

治療開始時、2週、4週、8週、12週、24週と本試験の受診毎に保存用の血液を採取し、エイズ治療・研究開発センター研究室で遠心分離後、EDTA Plasma 5ml を-80°のディープフリーザーで凍結保存する。研究に関連して更に詳しい情報が必要になった場合には、保存検体を使用して評価を行う。検体は研究IDによって管理する。本研究以外の目的で使用することはない。新たな研究で使用する場合には、再度、文書で同意を取得する。

|  |
| --- |

**8．試験薬投与の中止基準**

下記の場合に中止を検討する。

　　・QTcF 500>msec (心電図検査において)

・Ⅱ度/Ⅲ度房室ブロック(心電図検査において)

・肝不全の進行を認めた場合(Child-Pugh B or C)

・ALT＞ベースラインの5倍もしくは正常上限の10倍以上、総ビリルビンが正常上限2倍以上、INRが正常上限2倍以上

・血小板 2.5万/μL以下

・Grade 4の検査異常を認め、研究で使用した薬剤の関与が否定できない場合は治療薬を中止する

　　　・同意撤回の申し出があった場合

・試験薬投与を続けることが研究参加者の不利益となると研究者が判断した場合。

・何らかの理由で試験治療を継続できなくなった場合（例：嚥下困難、転院など）

**9．評価**

　　9.1 有効性の評価：持続的なウイルス学的反応

・HCV-RNAの治療後12週の時点で、HCV-RNAが検出感度未満を有効と判定する。

・ブレークスルー^*^、再燃^*^もしくはその両者でもないがウイルスを検出した場合をウイルス学的失敗とする。

＊ブレークスルー： 治療中に検出感度未満となったものが検出可能となる、もしくは　最低値から1log_10_の上昇を認めた場合

　＊再燃： 治療開始後12週（治療終了時）～治療開始後24週の間に、HCV-RNAが検出感度未満から検出可能となった場合。

　9.2 安全性の評価：Grade3以上の有害事象、または治療中断に至った有害事象の出現

**10．薬剤情報**

　　10.1ソホスブビル （商品名：ソバルディ）

　　　　核酸型のNS5Bポリメラーゼ阻害剤である。多くのHCV GTに対して抗ウイルス活性を有しており、in vitroの評価では、GT 1a、1b、2a、2b、3a、4a、5a、6aに対する効果を認めている。2015年3月にGT2型C型慢性肝炎・代償性肝硬変に対するソホスブビル/リバビリン併用療法が承認された。重度の腎機能障害(eGFR <30mL/分/1.73m^2^)または透析を必要とする腎不全の患者に対しては投与禁忌である。

　　併用禁忌薬：リファンピシン、カルバマゼピン、フェニトイン、セイヨウオトギリソウ

併用注意薬：リファブチン、フェノバルビタール

　　副作用：日本国内第3相臨床試験において73%の症例で発現した。その84%が軽度(grade 1)であった。最も高頻度の副作用は鼻咽頭炎の29%であり、他には貧血が12%、頭痛が10%、全身倦怠感が7%、皮膚掻痒が6%であった。Grade 4の副作用はなく、治療薬と関連したGrade 3の副作用は2例あり、1例が貧血、1例が高ビリルビン血症であった。副作用による中止例はなかった。

10.2　ダクラタスビル（商品名：ダクルインザ）

　　　　ダクラタスビルはNS5A阻害剤である。

　　　　併用禁忌薬：リファンピシン、リファブチン、フェニトイン、カルバマゼピン、フェノバルビタール、デキサメタゾン全身投与、セイヨウオトギリソウ

併用注意薬：アゾール系抗真菌剤、HIVプロテアーゼ阻害剤、コビシスタット含有剤、クラリスロマイシン、テラプレビル、エファビレンツ、ジゴキシン、ロスバスタチン、アトルバスタチン、フルバスタチン、シンバスタチン、ピタバスタチン、プラバスタチン

副作用：ダクラタスビル/アスナプレビル併用療法による国内第3相試験では、発現頻度の高かった有害事象は鼻咽頭炎、頭痛であった。検査異常値としてはAST/ALT上昇が見られ、Grade 3/4のALT上昇、AST上昇がそれぞれ7.2% (16例)、5.4% (12例)に出現した。投与中止例は10例(4.5%)であった。

　　10.3　ソホスブビル＋ダクラタスビル

　　　ソホソブビル＋ダクラタスビルによる海外の第3相試験（203例)では、有害事象は全身倦怠感（34例：17％）、嘔気（26例：13％）、頭痛（23例：11％）、下痢（15例：7％）、嘔吐（10例：5％）、皮疹（9例：4％）、不眠（8例：4％）、腹痛（7例：3%）、咳嗽（7例：3%）、めまい（6例：3%）、便秘（6例：3%）であった。有害事象により治療を中断した例は認めなかった。Grade 3/4の検査異常値はアタザナビル内服患者の総ビリルビンの上昇（8例：4％）、一過性のリパーゼの上昇（7例：3%）、INRの上昇（2例：1％）、ASTの上昇（1例：1％以下）であった。

**11．有害事象発現時の対応**

　　有害事象が発現した場合には、必要な診察、検査、処置を保険診療で適切に行う。中止基準に該当する場合、または中止が適当と判断される場合には、試験薬の投与を中止する。

**12．有害事象報告**

　　Grade 3以上の有害事象（グレードは添付資料を参考に判断する）または治療中断に至る有害事象が発現した場合には、直ちに研究代表者へ報告する。また、有害事象報告票（CRF-6）を作成し、研究グループで情報を共有する。以下に示す有害事象が発生した場合には、研究代表者は15日以内に「重篤な有害事象または不具合に関する報告書」を作成し、臨床研究推進室を通じて理事長と倫理委員会に報告する。また、予期しない重篤な有害事象が発生した場合には、有害事象への対応状況・結果を公表し、厚生労働大臣等に報告する。

　　重篤な有害事象

　　　・死に至るもの

　　　・生命を脅かすもの

　　　・治療のため入院または入院期間の延長が必要となるもの

　　　・永続的または顕著な障害・機能不全に陥るもの

　　　・子孫に先天異常を来すもの

　　　・上記以外の報告で重篤と判断されるもの

**13．対象に対する標準治療と研究の科学的合理性の根拠**

　　現在、日本国内ではGT3のC型肝炎に対する推奨治療薬がない。欧米では、GT3のC型肝炎に対してソホスブビル＋ダクラタスビルによる治療の有効性と安全性が報告されている^4)^。

**14．試験参加に伴って予想される利益と危険（不利益）**

　　14.1　予想される利益

　　　　本試験で用いるダクラタスビルはアスナプレビルとの併用でGT1のC型肝炎に対する治療薬として、ソホスブビルはレディパスビルとの併用でGT1およびリバビリンとの併用でGT2に対するC型肝炎に有効な治療薬として保険適用になっている薬剤である。また、GT3のC型肝炎に対しては、新しい組み合わせであるソホスブビル＋ダクラタスビルの治療が、海外でその有効性と安全性が明らかになっている。本試験の対象は、過去にインターフェロンが失敗した患者、またはインターフェロンの治療が不適だった患者である。GT3によるC型肝炎患者に対して、本試験薬を投与することにより、C型肝炎が治癒し、肝硬変の進行、肝癌の発生リスクを最小にできる可能性がある。

14.2　予想される危険（不利益）

　　本試験に参加することにより、試験に参加していない場合と比較して所定の検査および安全性確認のための受診、採血、心電図検査の回数が増える。

また、ソホスブビルとダグラタスビルの併用により、それぞれの副作用以上の予期せぬ有害事象の発現する可能性がある。これらの有害事象が発現した場合には、より頻回な診察と検査を行い、重症化を防ぐための適切な医療を行う。

**15．倫理的事項**

　　15.1患者の保護

　　　　本試験に関係するすべての研究者は、厚生労働省の「人を対象とする医学系研究に関する倫理指針」（平成26年12月22日）に従って本試験を実施する。

　　15.2インフォームド・コンセント

　　　　登録に先立って、研究責任医師または研究協力医師は、選択基準に合致する患者に対し、倫理委員会で承認が得られた説明同意文書を用いて本試験の概要を説明する。本試験に参加しなくても、診療上何ら不利益を受けないこと、試験参加後いつでも同意の撤回が可能なことを説明する。患者本人が試験参加の意思を示した場合には、文書による同意を得る。説明同意文書は原本をCRFと共に本試験の患者ファイルに保管し、コピーを患者に渡す。

　　15.3　代諾者からインフォームド・コンセントを受ける場合の手続き

　　　　本試験の対象者は、このままC型肝炎の治療を施さずにいると、確実に肝硬変が進行し、また、肝細胞癌を発症するリスクが非常に高い。そのため、インフォームド・コンセントを与える能力を欠く患者であっても、本試験参加の機会が他の感染者と同様に与えられるべきである。

成年であって、インフォームド・コンセントを与える能力を欠くと客観的に判断される可能性がある患者については、研究に携わらない2名以上により、能力を欠くことを確認し、代諾者からインフォームド・コンセントを受ける。本研究における代諾者は研究対象者の親に限定する（本パイロット試験では対象者が限定されているため代諾者を親に限定する）。代諾者に対しては、子が研究対象者となる理由とともに、説明同意文書を用いて研究の概要を説明する。代諾者への説明およびインフォームド・コンセントを受けた過程については、診療録に記録を残す。患者本人に対しては、患者が理解できる言葉を用いて平易に説明を実施する。

　　15.4 個人情報の取り扱い

　　　　試験参加者は、試験登録時に研究IDが付与され、以降カルテ番号と研究IDによる連結可能匿名化によりデータの管理を行う。匿名化対応表は、企画戦略局長に提出する。

**16．研究に係る試料及び情報の保管および廃棄の方法**

　　少なくとも、研究で収集した個人情報・個人データは、研究の終了について理事長に報告した日から5年を経過した日または研究の結果の最終の公表について理事長に報告した日から3年を経過した日のいずれか遅い日まで保管する。廃棄する場合は、印刷資料、電子媒体データなど、いずれの資料も、物理的に内容の読取りが不可能な状態にした後で廃棄する。書き換え可能な電子媒体については、ダミーデータを複数回上書きして元のデータを完全に復元不可能な状態にした後、適切に廃棄する。

| 研究に関連して収集された保存試料（EDTA Plasma）は、貴重なサンプルであるため保管期限は特に定めず、同意撤回がなされない限り保管を継続する。廃棄は、研究対象者が同意を撤回した場合に行う。廃棄は、検体を連結不可能匿名化した後、通常の診療サンプルと同様の方法で廃棄する。 |
| --- |

**17．研究機関の長への報告内容及び方法**

　　以下に定める事項が発生した場合には、規定の様式に則り臨床研究推進室を通じて理事長に報告する。研究の進捗状況は年1回、8月末までに定期報告する。

　　・重篤な有害事象が発生した場合

　　・研究を終了または中止した場合

　　・研究を中断または再開する場合

**18．研究の資金源と研究者の研究に係る利益相反**

　　本試験は、血友病HIV/HCV重複感染者に対する救済の一環であり、国立国際医療研究センターACC運営交付金、治療研究事業費によって実施する。従って本試験に係る利益の衝突はない。

**19．研究に関する情報公開**

　　本試験の実施に先立ち、研究概要をUMIN-CTRへ登録し、情報公開を行う。

**20．研究参加者の費用負担、謝礼**

　　本試験で投与されるソホスブビルおよびダグラタスビルの薬剤費および試験として実施される臨床検査の費用は血友病被害者救済のためのACC運営交付金、治療研究事業費より支出する。HCV治療に関する患者の費用負担はない。研究参加者に対する謝礼の支払いはない。

**21. 本試験によって生じた健康被害に対する補償の有無**

　　本試験で生じた健康被害に対する補償はない。賠償については、賠償責任保険に加入している医師を研究協力医師とし、当該保険によって対応する。上記で救済されるか否かに関わらず、通常の保険診療の範囲で適切な処置を行う。

**22. モニタリングと監査**

　　試験が安全にかつ研究実施計画書に従って実施されているかを確認する目的で、原則として、患者登録から1か月の時点で、症例ごとに全例モニタリングを実施する。また、試験薬投与終了時に全例モニタリングを実施する。モニタリングは22.1の内容を含み、原資料の直接閲覧によって実施する。研究代表者は研究協力者の中からモニターを指名する。モニターは、モニタリング実施後速やかにモニタリング報告書を作成し、研究代表者に報告する。重大な違反、逸脱があった場合には、研究代表者は理事長へ報告する。

　　監査は行わない。

　　22.1　モニタリングの項目

　　　　1）文書による同意取得と説明同意文書の保管

　　　　2）患者選択基準の適格性

　　　　3）有害事象発生の有無と報告状況

　　　　4）研究実施計画書逸脱の有無

　　　　5）その他、試験の進捗や安全性に関する問題の有無

　　　　6）症例報告書記入データと原資料との照合

　　　　7）試験関連保管文書および患者ファイルの保管状況

**23．研究組織**

　　23.1　研究代表者

　　　　　岡　慎一

国立国際医療研究センター

エイズ治療・研究開発センター　センター長

〒162-8655　東京都新宿区戸山1-21-1

TEL：03-5273-5193　　FAX：03-5273-5193 　e-mail：oka@acc.ncgm.go.jp

23.2　研究協力者

　　　　　菊池　嘉　　エイズ治療・研究開発センター　臨床研究開発部長

　　　　　潟永博之　　エイズ治療・研究開発センター　治療開発室長

　　　　　溝上雅史　　肝炎・免疫研究センター　センター長

　　　　　柳瀬幹雄　　第二消化器内科　医長

　　　　　小形幹子　　エイズ治療・研究開発センター　研究助手

　　　　　高野　操　　臨床研究試料管理室　CRC

　　23.3　患者の問い合わせ窓口

　　　　　岡　慎一

国立国際医療研究センター

エイズ治療・研究開発センター　センター長

〒162-8655　東京都新宿区戸山1-21-1

TEL：03-5273-5193　 e-mail：oka@acc.ncgm.go.jp

**24．参考文献**

1. 日本肝臓学会編 C型肝炎治療ガイドライン(第3.5版)

2. Vogel M et al. Acute hepatitis C infection in HIV-positive patients. *Curr Opin Infect Dis* 24:1-6, 2011.

3. Brau N et al. Presentation and outcome of hepatocellular carcinoma in HIV-infected patients: U.S.-Canadian multicenter study. *J Hepatol* 47:527-37, 2007.

4. D.L Wyles et al. Daclatasvir plus Sofosbuvir for HCV in patients co-infected with HIV-1. *N Engl J Med* 373(8):714-725, 2015.

5．Lacombe K et al. Daclatasvir plus Sofosbuvir with or without ribavirin in patients with HIV-HCV coinfection: Interim analysis of a French multicenter compassionate use program. A1444-258 study IAS 2015 Jul 19-22, Vancouver
